# Safety Evaluations of *Bifidobacterium bifidum* BGN4 and *Bifidobacterium longum* BORI

**DOI:** 10.3390/ijms19051422

**Published:** 2018-05-09

**Authors:** Min Jeong Kim, Seockmo Ku, Sun Young Kim, Hyun Ha Lee, Hui Jin, Sini Kang, Rui Li, Tony V. Johnston, Myeong Soo Park, Geun Eog Ji

**Affiliations:** 1 Research Center, BIFIDO Co., Ltd., Hongcheon 25117, Korea; minjeong.kim@bifido.com (M.J.K.); kimwho0222@daum.net (S.Y.K.); hyunha_92@daum.net (H.H.L.); 2 Fermentation Science Program, School of Agribusiness and Agriscience, College of Basic and Applied Sciences, Middle Tennessee State University, Murfreesboro, TN 37132, USA; seockmo.ku@mtsu.edu (S.K.); tony.johnston@mtsu.edu (T.V.J.); 3 Department of Food and Nutrition, College of Human Ecology, Seoul National University, Seoul 08826, Korea; jh1030@snu.ac.kr (H.J.); kangsini89@naver.com (S.K.); iouljt@snu.ac.kr (R.L.); 4 Department of Hotel Culinary Arts, Yeonsung University, Anyang 14001, Korea

**Keywords:** probiotics, safety, antibiotics resistance, functional foods, nutraceuticals

## Abstract

Over the past decade, a variety of lactic acid bacteria have been commercially available to and steadily used by consumers. However, recent studies have shown that some lactic acid bacteria produce toxic substances and display properties of virulence. To establish safety guidelines for lactic acid bacteria, the Food and Agriculture Organization of the United Nations (FAO)/World Health Organization (WHO) has suggested that lactic acid bacteria be characterized and proven safe for consumers’ health via multiple experiments (e.g., antibiotic resistance, metabolic activity, toxin production, hemolytic activity, infectivity in immune-compromised animal species, human side effects, and adverse-outcome analyses). Among the lactic acid bacteria, *Bifidobacterium* and *Lactobacillus* species are probiotic strains that are most commonly commercially produced and actively studied. *Bifidobacterium bifidum* BGN4 and *Bifidobacterium longum* BORI have been used in global functional food markets (e.g., China, Germany, Jordan, Korea, Lithuania, New Zealand, Poland, Singapore, Thailand, Turkey, and Vietnam) as nutraceutical ingredients for decades, without any adverse events. However, given that the safety of some newly screened probiotic species has recently been debated, it is crucial that the consumer safety of each commercially utilized strain be confirmed. Accordingly, this paper details a safety assessment of *B. bifidum* BGN4 and *B. longum* BORI via the assessment of ammonia production, hemolysis of blood cells, biogenic amine production, antimicrobial susceptibility pattern, antibiotic resistance gene transferability, PCR data on antibiotic resistance genes, mucin degradation, genome stability, and possession of virulence factors. These probiotic strains showed neither hemolytic activity nor mucin degradation activity, and they did not produce ammonia or biogenic amines (i.e., cadaverine, histamine or tyramine). *B. bifidum* BGN4 and *B. longum* BORI produced a small amount of putrescine, commonly found in living cells, at levels similar to or lower than that found in other foods (e.g., spinach, ketchup, green pea, sauerkraut, and sausage). *B. bifidum* BGN4 showed higher resistance to gentamicin than the European Food Safety Authority (EFSA) cut-off. However, this paper shows the gentamicin resistance of *B. bifidum* BGN4 was not transferred via conjugation with *L. acidophilus* ATCC 4356, the latter of which is highly susceptible to gentamicin. The entire genomic sequence of *B. bifidum* BGN4 has been published in GenBank (accession no.: CP001361.1), documenting the lack of retention of plasmids capable of transferring an antibiotic-resistant gene. Moreover, there was little genetic mutation between the first and 25th generations of *B. bifidum* BGN4. Tetracycline-resistant genes are prevalent among *B. longum* strains; *B. longum* BORI has a *tet*(W) gene on its chromosome DNA and has also shown resistance to tetracycline. However, this research shows that its tetracycline resistance was not transferred via conjugation with *L. fermentum* AGBG1, the latter of which is highly sensitive to tetracycline. These findings support the continuous use of *B. bifidum* BGN4 and *B. longum* BORI as probiotics, both of which have been reported as safe by several clinical studies, and have been used in food supplements for many years.

## 1. Introduction

Since “probiotics” first emerged in the 1960s [1], the term has been defined by various scholars and groups. In recent years, probiotics have been clearly defined by several regulatory organizations [2]. According to the FAO/WHO, probiotics can be defined as “live microorganisms which, when administered in adequate amounts, confer a health benefit to the host” [3]. Other experts similarly define probiotics as “live microorganisms which, when ingested or locally applied in sufficient numbers, provide the consumer with one or more proven health benefits” [4]. Edible microorganisms regarded as probiotic bacteria are derived from various strains, species, and genera, which have been studied with regard to various human health benefits [5]. A variety of microorganisms, including *Bacillus* spp., *Lactobacillus* spp., *Bifidobacterium* spp., *Streptococcus* spp., and *Propionibacterium* spp., are regarded as probiotics, and are known to be involved in the vitamin biosynthesis of the host’s nutrition metabolism and physiological function via immune-mediated effects [6,7]. Of these probiotic microorganisms, *Lactobacillus* spp. and *Bifidobacterium* spp. have been utilized globally in fermented food products and commercially-produced food supplements [8]. As of July 2010, the genomic sequences of approximately 11 *Bifidobacterium* and 21 *Lactobacillus* species have been completely analyzed, whose microbial genomic sequences offer exact evidence of the probiotics’ genera and species [9]. Some experts have found that consumer demand for food or food supplements containing lactic acid bacteria have led to the exponential growth of healthy trends in the global food market [10]. However, this phenomenon cannot disregard microbial safety standards or allow lactic acid bacteria to be used indiscriminately without scientific research or safety verification [3,11]. Also, a probiotic safety assessment should consider the probiotic’s physiological characteristics, treatment method (e.g., oral administration, skin spray, gel, capsule, etc.), exposure dosage, consumers’ health, and the physiological functions required for effective probiotic performance [12].

In 2002, the FAO created four basic guidelines for food industry probiotic application, because a variety of commercially-available microorganisms had been sold to consumers as probiotics without clear labeling standards. The FAO guidelines summarized by Huys et al. [13] are as follows: (i) “the assessment of strain identity (i.e., genus, species, and strain level); (ii) in vitro tests to screen potential probiotic strains (e.g., resistance to gastric acidity, bile acid, and digestive enzymes, antimicrobial activity against potentially pathogenic bacteria, etc.); (iii) assessment of safety: requirement of proof that a probiotic strain is safe and without contamination in its delivery form; and (iv) in vivo studies for the substantiation of the health effects in the target host”. In addition, the FAO recommended that various tests (e.g., analysis of antibiotic resistance, metabolic activity, toxin production, hemolytic activity, infectivity in immune-compromised animal models, human side effects, and adverse outcomes in consumers) be conducted with the probiotic microorganisms to demonstrate their safety to hosts and elaborate on section three of the aforementioned guidelines [3]. However, these safety assessment items are recommendations rather than legal requirements. Various research groups have evaluated the safety of probiotic bacteria according to their cell types and microbial functionalities by incorporating additional experimental methods [14,15,16]. In 2002, the European Union Scientific Committee on Animal Nutrition issued guidelines for the safety assessment and regulation of edible microorganisms utilized in food and animal feeds. The corresponding “qualified presumption of safety (QPS)” guidelines from 2016 are as follows [11]: (1) definition of the taxonomy of the microbe; (2) collection of sufficient information providing the basis for QPS status, including any scientific literature, history of use, industrial applications, ecological data, and human intervention data; (3) exclusion of pathogenicity; and (4) definition of the end use. Based on this guideline, QPS status may be granted to probiotic cells in European Union food markets if there are no safety problems with a particular taxon or if the safety problem is alleviated. It is commonly agreed that microbial safety should demonstrate the (i) species characteristics with genetic information, (ii) phenotypic evidence, (iii) isolation history, (iv) absence/presence of antibiotic-resistant properties, and (v) potential virulence and/or pathogenic factors [17].

One of the greatest safety concerns for commercially-produced lactic acid bacteria is that some of the microorganisms supplied in the form of diets may act as the donor of antibiotic-resistant plasmids to intestinal pathogens [18,19]. Several reports have found that in the presence of antibiotic treatment, some strains survive in the human gastrointestinal tract due to the transferred resistance of plasmids [20,21,22]. A variety of microbial genes can be transferred to enteric bacteria in the intestine via plasmids, resulting in the spread of antibiotic-resistance [23]. Therefore, ensuring the safety of a probiotic strain is necessary prior to the mass production of lactic acid bacteria for commercial purposes.

Although some *Bifidobacterium* and *Lactobacillus* spp. have shown promise in in vivo and in vitro studies, there is a lack of clear clinical evidence to support the health benefits of these microorganisms [24]. Therefore, many groups and researchers are trying to prove the efficacy of lactic acid bacteria through clinical experimentation. *B. bifidum* BGN4 and *B. longum* BORI were isolated from the feces of healthy breast-feeding infants, and have been commercially used as food ingredients since 2000 [25,26,27,28,29]. Some bifidobacteria strains, including *B. bifidum* and *B. longum*, are registered as functional ingredient, Probiotics (II.2.51) in Health Functional Food Code of Korea [30]. Over the years, many studies have revealed the functionalities of *B. bifidum* BGN4 [28], and its complete genomic sequence was reported to GenBank [31]. *B. longum* BORI, also isolated from a healthy breast-fed infant and deposited in KCCM (Korean Culture Center of Microorganisms, 14092), was proven to statistically shorten the duration of diarrhea in a clinical study of infants infected with rotavirus [25]. Both probiotic strains have been proven to effectively form healthy intestinal microflora without any adverse effects. However, further systematic research should be conducted to prove their safety for academic and commercial applications. The aim of this study was to validate the safety of *B. bifidum* BGN4 and *B. longum* BORI by conducting FAO/WHO recommended experiments and other published safety research.

## 2. Results and Discussion

### 2.1. Ammonia Production

Intestinal bacteria can degrade various nitrogen sources (e.g., proteins, peptides, and amino acids) present in the feces of the intestinal track [32]. These naturally-occurring microbiota and artificially-administered flora have the potential to produce various toxic substances during the deamination stage via nitrogen derivatives. Multiple potentially toxic products (i.e., phenol, ammonia, and indole [33], are possible products of the proteolytic process, especially in the large intestine. Thus, bacterial ammonia production is highly relevant to human intestinal health, and a necessary component of the evaluation to demonstrate the safety of commercial probiotics. Moreover, recent studies have also shown that ammonia produced by gut microorganisms can affect the liver and act as a cofactor in chronic liver damage. Vince and Burridge [34] reported that considerable amounts of ammonia were generated by the Gram-negative anaerobes, *Clostridia* (including *Clostridium perfringens*), *Enterobacter*, and *Bacillus* spp. Some strains of streptococci, micrococci, and Gram-positive non-spore forming anaerobes produced moderate concentrations of ammonia. By contrast, Gram-positive aerobic rods, in particular *Lactobacilli*, produced very little ammonia.

The ammonia production of *B. bifidum* BGN4 and *B. longum* BORI were assessed to verify the safety of these probiotics. In this study, *B. bifidum* BGN4, *B. longum* BORI, and other probiotic strains did not produce ammonia. By contrast, *Bacteroides* spp., *Clostridium perfringens,* and *Enterobacter* spp., which are known harmful bacteria and used as positive controls in this study, produced 12.9 ± 1.3 to 161.0 ± 6.6 μg/mL of ammonia (Table 1). This test included three replications, and the values presented are the means ± the standard deviations. This study found no indication of the production of ammonia by *B. bifidum* BGN4 and *B. longum* BORI.

### 2.2. Hemolytic Property Test

**Figure 1:**
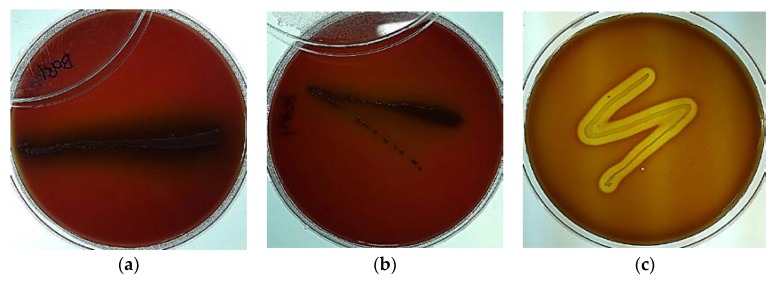
*B. bifidum* BGN4 ((**a**); back light) and *B. longum* BORI ((**b**); back light) growth with no blood cell lysis. Complete lysis of red blood cells was observed, with clear zones around the *Listeria ivanovii* subsp. *ivanovii* ATCC 19119 colonies ((**c**); positive control, back light).

The 2002 FAO/WHO Guidelines on Probiotics Safety Considerations clearly states that “if the strain under evaluation belongs to a species with known hemolytic potential, determination of hemolytic activity is required” [3]. Microbial hemolysis properties are a common concern for pathogenic bacteria (e.g., enterococci, and streptococci) because of the potential for anemia and edema in the host. Although *Bifidobacterium* spp. are normal, naturally-occurring intestinal microbiota that have been widely included in functional foods and utilized by nutraceutical industries, they can potentially behave as opportunistic pathogenic microorganisms similar to common commensal microorganisms. Therefore, hemolysis assay tests should be conducted on potential probiotic bacteria. Visualizing the physical changes caused by hemolytic activity by culturing the microorganisms on a medium containing animal or human blood is a commonly used tool to evaluate the hemolytic properties of pathogens. In this study, the potential hemolytic activity of *B. bifidum* BGN4 and *B. longum* BORI were assessed using the blood agar plating method. *Listeria ivanovii* subsp. *ivanovii* ATCC 19119 (positive control) showed β-hemolysis colorless zones around the cell colonies, whereas *B. bifidum* BGN4 and *B. longum* BORI showed no hemolysis and no change of color in the periphery of the colonies (Figure 1).

### 2.3. Evaluation of Biogenic Amine Production

Biogenic amines (e.g., cadaverine, histamine, tyramine, and putrescine) have hydrophobic skeletons and naturally-occurring organic polycation molecules derived from the amino acids in animals and humans [35]. These molecules are involved in multiple metabolic and intracellular activities of mammals (e.g., synaptic transmission, blood pressure control, allergic response, and cellular growth control). Traditionally, a variety of probiotic bacteria have been artificially integrated into fermented foods, due to their beneficial effects and flavor-enhancing properties [36]. Their biogenic amine levels have been regarded as an indicator of microbial activity and food freshness due to the fact that biogenic amines are generated via microbial metabolic activities (i.e., decarboxylation and the transamination of protein molecules) [37]. While biogenic amines are commonly found in fresh meat, vegetables, and cheese, ingestion of large amounts of biogenic amines may cause symptoms in humans and animals that are similar to severe allergic reactions [38]. One of the most common issues in the probiotics field in recent years has been whether probiotics contribute to the production of biogenic amines, and how they contribute to the production of biogenic amines [15]. Complex biogenic amines (i.e., polyamines having more than one amino group) were initially thought to be naturally present in a variety of fresh foods, but recent studies have shown that these chemicals can accumulate as a result of microbial activity. Some edible microorganisms and probiotic strains were reported to produce biogenic amines [39,40,41]. Therefore, the aim of this study was to examine the biogenic amine production of *B. bifidum* BGN4 and *B. longum* BORI as a component of an overall probiotic safety evaluation. The biogenic amine content of the bifidobacteria is featured in Table 2.

The biogenic amine content of these strains was derived by subtracting the background content of the biogenic amines in each medium. *B. bifidum* BGN4 and *B. longum* BORI did not produce cadaverine, histamine, or tyramine; however, they produced 24.23 and 16.58 µg/mL of putrescine, respectively. The levels produced were not of concern. Putrescine is a natural substance present in various foods [42,43,44]. Putrescine, also naturally found in small amounts in living cells, is formed by the decarboxylation of ornithine and arginine. It is also a metabolite produced by various edible probiotic cells. Putrescine is also a precursor of spermidine and spermine. The polyamines putrescine, spermidine, spermine, and cadaverine are essential components of living cells, and play an important role in the formation of nucleic acid, protein synthesis, and membrane stability. Of the various biogenic amines detected in a variety of fruits, juices, and vegetables, putrescine was the most common. Kalač [42] reported that putrescine was commonly found in frozen spinach puree (average 12.9 mg/kg), ketchup (average 52.5 mg/kg), concentrated tomato paste (average 25.9 mg/kg), and frozen green pea (average 46.3 mg/kg). The putrescine content of fermented foods and beverages [43] was found to be 9 mg/kg (3–25 mg/kg, *n* = 28) in sherry, 154 mg/kg (6–550 mg/kg, *n* = 8) in sauerkraut, 19 mg/kg (1–71 mg/kg, *n* = 8) in Dutch cheese, and 52 mg/kg (1–190 mg/kg, *n* = 14) in fermented sausage. Furthermore, the putrescine found in the traditional cheeses made from ewe’s whole milk in Sardinia, Italy, increased to 1658 mg/L during ripening [44]. *Bifidobacterium* spps. (i.e., *Bifidobacterium* CCDM 94, *B. adolescentis* CCDM 223, *B. animalis* ssp. *lactis* CCDM 239, 240, 241, and 374, *B. bifidum* CCDM 559, and *B. longum* CCDM 569) are known to produce cadaverine, putrescine, tyramine, and spermidine [41]. According to Pollark et al. [45], putrescine is contained in human breast milk (0~3804 nmol/L) and commercial formula milk (0~1057 ± 25 nmol/L).

Therefore, it matters how much putrescine occurs naturally. Some researchers theorize that putrescine in food is likely to show synergistic effects on histamine toxicity. However, such synergy has not been proven or reported with experimental data, as far as we know. Moreover, the European Food Safety Authority (EFSA) [46] also identified a lack of research to identify the exact levels of putrescine required to increase the side effects of histamine. *B. bifidum* BGN4 and *B. longum* BORI did not produce any cadaverine, histamine, or tyramine during the fermentation process. *B. bifidum* BGN4 and *B. longum* BORI produced low levels of putrescine, which was also found in both media (i.e., whole milk medium = 24.43 µg/mL, *B. bifidum* BGN4 culturing medium (whole milk) = 48.67 µg/mL, MRS medium = 26.60 µg/mL, *B. longum* BORI culturing medium (MRS) = 43.17 µg/mL). The human oxidation system of mono-amine and diamine oxidase includes small amounts of biogenic amines that are usually metabolized and harmless, because humans and animals have the ability to decompose them in vivo.

### 2.4. Antimicrobial Susceptibility and Antibiotic Resistance Transferability

#### 2.4.1. Antibiotic Susceptibility

Various lactic acid bacteria research groups have warned that some lactic acid bacteria consumed as food or feed may have antibiotic-resistant properties. Since this resistance capability could be transferred to other pathogens via plasmids, the assessment of antibiotic resistance is an important criterion for evaluating the safety of strains used in food and feed [47]. Moreover, the acquired transferable genes have been characterized in bifidobacteria and lactobacilli [48]. In order to distinguish antibiotic-resistant from antibiotic-susceptible microorganisms, the EFSA has established microbiological cut-off values for the antibiotic-resistance of microorganisms used as food and/or feed additives. These microbiological cut-off values were determined based on the distribution of the chosen antimicrobials’ minimum inhibitory concentrations (MICs) in cell populations belonging to a single taxonomical unit [49].

All *Bifidobacterium* spp. in this study were susceptible to ampicillin, chloramphenicol, clindamycin, erythromycin, penicillin G, rifampicin, and vancomycin (MIC ranging from 0.01 to 4 μg/mL) and generally resistant to aminoglycoside antibiotics, such as gentamicin, kanamycin, neomycin, and streptomycin (MIC ranging from >32 μg/mL, Table 3). The MIC values of *B. bifidum* BGN4 and *B. longum* BORI, with the exception of gentamicin and tetracycline, were equal to or lower than the established EFSA cut-off values suggested by the EFSA’s Panel on Additives and Products or Substances used in Animal Feed (FEEDAP) [49]. The susceptibility tendencies of *B. bifidum* BGN4 and *B. longum* BORI were similar to other studies [50,51,52], with the exception of high MIC to tetracycline in *B. longum* BORI. Penicillin G, ampicillin, vancomycin, gentamicin, erythromycin, trimethoprim–sulfamethoxazole, and metronidazole are known as frequently used antibiotics in pediatric patients [53]. *B. bifidum* BGN4 and *B. longum* BORI are resistant to trimethoprim–sulfamethoxazole but six of ten *Bifidobacterium* spp. strains also showed MIC values over 128 μg/mL in this research (Table 3).

Mättö et al. [54] reported *Bifidobacterium* strains displayed generally high MICs for streptomycin and gentamicin, and suggested their resistances were intrinsic. Ammor et al. [48] isolated probiotic bacteria from 21 food samples, such as yogurt, yogurt-type fermented milk, and pharmaceutical products, and found 22 strains of *Bifidobacterium* spp. In their study, Bifidobacteria were resistant to aminoglycoside (MIC_90_ ranges from 64 to 1000 μg/mL) and strongly resistant to kanamycin (MIC_90_ = 1000 μg/mL). They also demonstrated that some MIC ranges did not overlap, implying that the antibiotics related to these MIC ranges are usable as ingredients in selective media. They suggested the selective range of gentamicin was from 32 to 64 μg/mL and kanamycin was 64 to 500 μg/mL for *Bifidobacterium*. Therefore, gentamicin containing medium [55] and mupirocin containing medium [56,57] have been used for the selection and enumeration of *Bifidobacterium*. Accordingly, this resistance could be considered as intrinsic. Antibiotic resistance transferability studies were conducted to confirm the nature of this resistance.

#### 2.4.2. Antibiotic Resistance Transferability

Since *B. bifidum* BGN4 and *B. longum* BORI showed high antibiotic resistance to gentamicin and/or tetracycline in these antimicrobial susceptibility tests, tetracycline resistance transferability tests were conducted using *L. fermentum* AGBG1, a recipient strain that is highly susceptible to tetracycline. In order to test the transferability of gentamicin resistance of *B. bifidum* BGN4 and *B. longum* BORI, *L. acidophilus* ATCC 4356 was used as a recipient strain, due to its high gentamicin sensitivity. The conjugation results are shown in Table 4.

*L. fermentum* AGBG1 did not grow when cultured alone or co-cultured with *B. longum* BORI in the media containing tetracycline. The antimicrobial susceptibility test reported herein found that while *B. bifidum* BGN4 was very susceptible to tetracycline (MIC 1.0 μg/mL), *B. longum* BORI was resistant to tetracycline (MIC 64 μg/mL). However, the tetracycline resistance of *B. longum* BORI was not transferred to the recipient, *L. fermentum* AGBG1, in this study. *L. acidophilus* ATCC 4356, which is highly susceptible to gentamicin, grew well in normal MRS medium; however, *L. acidophilus* ATCC 4356 did not grow in the MRS medium containing gentamicin or the media that was co-cultured with *B. bifidum* BGN4 or *B. longum* BORI. By contrast, *B. bifidum* BGN4 and *B. longum* BORI showed resistance to 64 μg/mL gentamicin in this study. Therefore, this proves *B. bifidum* BGN4′s resistance to gentamicin and *B. longum* BORI’s resistance to gentamicin and tetracycline were not transferred to the recipient strains. It is worth noting that a 2011 report published by the Agency for Healthcare Research and Quality (AHRQ) [58] extensively reviewed 622 studies on six genera (i.e., *Lactobacillus*, *Bifidobacterium*, *Saccharomyces*, *Streptococcus*, *Enterococcus*, and *Bacillus* spp.), and found no clinical evidence of the theoretical possibility of gene transfer from probiotics to other microorganisms.

#### 2.4.3. PCR Results on Antibiotic Resistance Genes

**Figure 2:**
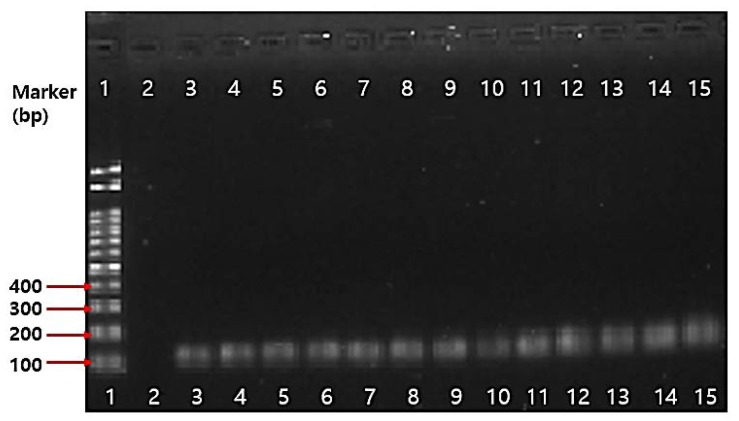
PCR analysis results of various *Bifidobacterium* spp.: Lane 1: marker; Lane 2: without loading; Lane 3: *B. lactis* AS60; Lane 4: *B. bifidum* KCTC 3440; Lane 5: *B. longum* BORI; Lane 6: *B. longum* KCCM 91563; Lane 7: *B. lactis* BB-12; Lane 8: *B. longum* RD47; Lane 9: *B. bifidum* BGN4; Lane 10: *B. thermophilum* KCCM 12097; Lane 11: *B. adolescentis* ATCC 15703; Lane 12: *B. lactis* AD011; Lane 13: *B. infantis* ATCC 15697; Lane 14: *B. breve* M-16V; Lane 15: *B. animalis* ATCC 25527.

**Figure 3:**
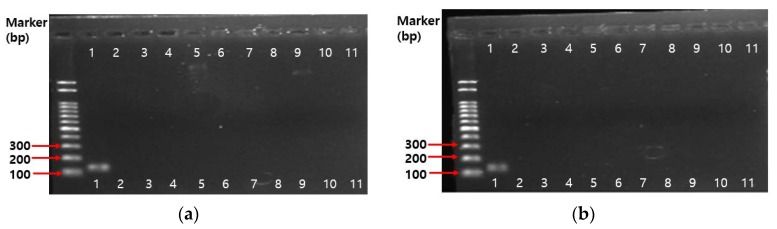
PCR analysis results of the antibiotic resistance gene in *B. bifidum* BGN4 and *B. longum* BORI: (**a**) *B. bifidum* BGN4; (**b**) *B. longum* BORI; Lane 1: *Bifidobacterium* genus-specific primers; Lane 2: gentamicin(*aaac(6)-aph(2)*), Lane 3: kanamycin(*AphA3*), Lane 4: streptomycin(*aadE*), Lane 5: trimethoprim(*dfrA*); Lane 6: tetracycline K(*tet*(K)); Lane 7: tetracycline L(*tet*(L)); Line 8: tetracycline M(*tet*(M)), Lane 9: tetracycline O(*tet*(O)), Lane 10: tetracycline S(*tet*(S)); Lane 11: kanamycin(*aaaD*).

Even though the whole genome of *B. bifidum* BGN4 (Accession no.: CP001361.1) and *B. longum* BORI show that neither contain a plasmid capable of transferring the antibiotic-resistance gene, PCR analysis on ten antibiotic genes such as gentamicin(*aaac(6)–aph(2)*), kanamycin(*AphA3, aaaD*), streptomycin(*aadE*), trimethroprim(*dfrA*), and tetracycline(*tet*(K)*, tet*(L)*, tet*(M)*, tet*(O), *tet*(S)) were conducted. All the tested *Bifidobacterium* spp. in this study were identified using 16S rRNA *Bifidobacterium* genus specific primers (Figure 2). The PCR results on antibiotics genes are shown in Figure 3. There were no amplicons that indicate resistance genes in *B. bifidum* BGN4, *B. longum* BORI, and other *Bifidobacterium* spp. in this study.

Recently, the intrinsic gentamicin-resistance of *Bifidobacterium* spp. was putatively attributed to the presence of two genes, namely *Bbr_0651* and *Bbr_1586*, which are enzymes present in the *Bifidobacterium* chromosome DNA, with both coding for putative phosphotransferase enzymes [59]. Tetracycline resistance genes (*tet*) are widely distributed in the *Bifidobacterium* genus; however, it is known as a ribosomal protection protein [48,60]. The tetracycline W (*tet*(W)) gene was found in *B. longum* BORI chromosome DNA. In the study of Mättö et al. [54], human- and probiotic-associated *Bifidobacterium* species (203 strains) showed high MIC values for tetracycline (i.e., ≥16 mg/mL; prevalence of 4–18%) that were attributed to the presence of tetracycline genes (*tet*), where *tet*(W), and *tet*(O) were detected. The *tet*(W), and *tet*(M) were found in 26, and 7%, respectively, of the *Bifidobacterium* isolates. The role of the *tet*(W) gene is presumed to be the translation factor GTPase of the TRAFAC family, which induces a noncovalent modification to the ribosome that destroys the effect of tetracycline, inhibiting protein synthesis [61].

### 2.5. Mucin Degradation

The intestinal mucus gel layer is an important constituent of the intestinal barrier that consists of a glycoprotein family. Multiple groups have reported that bacterial translocation can occur in infants and immunocompromised hosts, even if the intestinal mucus acts as a biological shield from microbes. This bacterial translocation has the potential to cause sepsis, and is one of the most serious probiotic safety concerns. Some scientists have also reported the possibility of bacteremia—endocarditis due to the administration of probiotic strains [62,63]. According to Ruas-Madiedo et al. [64], some *Bifidobacterium* spp. demonstrate mucolytic activities and have genes that induce mucin degrading enzymes. However, the majority of *Bifidobacterium* spp., such as *B. longum* and *B. pseudocatenulatum*, did not display mucolytic activity.

**Figure 4:**
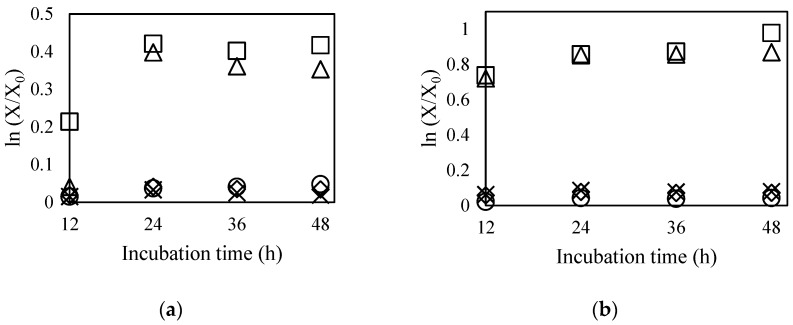
Growth curves of *B. bifidum* BGN4 (**a**) and *B. longum* BORI (**b**) in modified MRS with various carbon sources: basal medium (glucose-free MRS, ◇), basal medium with 0.5% mucin (×), 1.0% mucin (⚪), 0.5% glucose (Δ), and 1.0% glucose (□).

In order to confirm their microbial safety, it is necessary to evaluate translocation ability via mucolytic capacity analysis of each strain. In this study, the translocation capabilities of *B. bifidum* BGN4 and *B. longum* BORI were measured using in vitro mucolytic assays. The cell growth rates after incubation were examined in five kinds of modified MRS media by measuring their absorbances at 550 nm: basal medium (glucose-free MRS, ◇), basal medium with 0.5% mucin (×), 1.0% mucin (⚪), 0.5% glucose (Δ), and 1.0% glucose (□) (Figure 4).

In general, when simple sugars (e.g., glucose, fructose, maltose, and sucrose) are added, mucinase production can be inhibited due to catabolic repression. A false negative result can be obtained despite the microorganisms’ potential to produce mucinolytic enzymes. Therefore, to obtain accurate data, glucose, which is generally used as a carbon source in the MRS medium, was intentionally removed from the medium in which the experimental cells were cultivated. If *B. bifidum* BGN4 and *B. longum* BORI were able to produce mucinase, they would be able to source carbon and grow actively through mucin digestion. As shown in Figure 2, the growth of both probiotic strains was actively induced when glucose was added as a carbon source. However, when mucin was added instead of glucose, no growth was observed in either strain. These observations clearly indicate that *B. bifidum* BGN4 and *B*. *longum* BORI did not use mucin as a carbon source for their growth. This study, as suggested by other studies [65,66], shows that neither *B. bifidum* BGN4 nor *B. longum* BORI degrade mucin, indicating that the strains are not capable of damaging intestinal surfaces and do not have translocational abilities.

### 2.6. Genetic Stability

The genetic variation of edible microorganisms possibly results in indels (i.e., gene deletion and insertion) and mutations. A critical consideration of commercializing probiotics is whether it is possible to maintain genetic safety over the long term. However, the genetic stability of commercial probiotic strains has not yet been reported. Theoretically, an evaluation of genetic stability requires the entire genome sequence of the strain.

The entire genome sequence of *B. bifidum* BGN4 has been published [31], and consists of a 2,223,664 bp circular chromosome (62.65% G+C) with no plasmids. A total of 1835 coding sequences (CDSs), 7 pseudogenes, 3 rRNA operons, and 52 tRNAs were compiled from the nucleotide sequence. This study shows that the similarity in the genomic comparison between 1st generation and 25th generation samples were 99.9996~99.9998% via the Orthologous Average Nucleotide Identity (OrthoANI) value. (Table 5).

The difference between 0.0002% and 0.0004% is equivalent to 4.4 to 8.8 bp mutation of the entire nucleotide sequence, which can be assumed to be due to sequencing errors or spontaneous evolutionary mutations. Therefore, it is concluded that there was little genetic mutation, and the genetic information did not change in the process of cultivating 25 generations.

### 2.7. Virulence Factors

The genome sequences of *B. bifidum* BGN4 and *B. longum* BORI were compared with the genome sequences of four well-known pathogens (*E. coli*, *Enterococcus*, *Listeria*, and *Staphylococcus aureus*). The virulence factors included *E. coli* Shiga toxin gene and *S. aureus* exoenzyme genes, host immune alteration or evasion genes and toxin genes. No virulence factors were found in the genomic sequences of *B. bifidum* BGN4 and *B. longum* BORI. Thus, this result shows that the genomic sequences of *B. bifidum* BGN4 and *B. longum* BORI do not include toxic or pathogenic genes related to *E. coli*, *Enterococcus*, *Listeria*, and *S. aureus*.

## 3. Materials and Methods

### 3.1. Microorganisms

The bacterial strains, including origin, culture medium, and test methods used in this study are presented in Table 6.

### 3.2. Ammonia Production Test

*B. bifidum* BGN4, *B. longum* BORI, *B. breve* ATCC 15701, *L. plantarum* KFRI 708, *B. fragilis* ATCC 25285, *B. thetaiotaomicron* ATCC 29741, *C. perfringens* ATCC 13124, *E. cloacae* ATCC 13047, and *E. faecalis* ATCC 19433 were anaerobically cultured in brain heart infusion (BHI) (BD BBL™, Franklin Lakes, NJ, USA) media at 37 °C for 5 days. The production of ammonia by catalyzed indophenol reaction was determined according to the method of Chaney and Marbach [67]. To evaluate the generated extracellular ammonia levels, the media supernatants of each strain were obtained by centrifuging at 10,000× *g* at 4 °C for 30 min. The media was then adjusted to pH 7 using 1 N NaOH. Two solutions were prepared as follows: Solution 1 consisted of 2 g phenol and 0.01 g sodium nitroferricyanide dehydrate dissolved in 200 mL distilled water and Solution 2 consisted of 1 g sodium hydroxide and 0.08 g sodium hypochlorite dissolved in 200 mL distilled water. Aliquots (10 μL) of Solutions 1 and 2 were added to 96 well plates with 100 μL of the media supernatants of each strain. Three replications of this test were conducted on each strain. The 96 well plates were maintained at room temperature for one hour, and the absorbance was measured at 625 nm. Bacteria-free BHI medium was used as a negative control and the ammonia concentration was calculated using a standard curve.

### 3.3. Hemolytic Test

*B. bifidum* BGN4 and *B. longum* BORI were anaerobically cultured in blood agar (BHI broth medium supplemented with 1.5% agar and 5% sheep blood) at 37 °C for 2 days. *Listeria ivanovii* subsp. *ivanovii* ATCC 19119, a positive control for hemolysis, was aerobically cultivated in blood agar at 37 °C for 2 days. The plates were then analyzed for the presence or absence of microbial hemolysis properties by holding the plate up to a light source and viewing through both sides of the plate. Strains that produced green-hued zones around the colonies (α-hemolysis) or did not produce any hemolysis on the blood plates (γ-hemolysis) were considered non-hemolytic. Strains that displayed blood lyses zones (white-hued zones) around the colonies were classified as microorganisms with hemolytic (β-hemolysis) properties.

### 3.4. Biogenic Amine Production Test

*B. bifidum* BGN4 and *B. longum* BORI were anaerobically cultured in whole milk (Seoul Milk, Korea) or de Man–Rogosa–Sharpe (MRS) broth (BD Difco™, Franklin Lakes, NJ, USA)) with supplementation of 0.05% (*w*/*w*) l-cysteine-HCl (Sigma, St. Louis, MO, USA) at 37 °C for 15 h. Four biogenic amines (cadaverine (≥97.0%, Cat. #33211), histamine (≥97.0%, Cat. #H7125), putrescine (≥98.5%, Cat. #51799), and tyramine (99%, Cat. #T90344)) were purchased from Sigma-Aldrich (St. Louis, MO, USA). 1,7-Diaminoheptane (internal standard; ISTD, 98%, Cat. #D174708), dansyl chloride (≥99.0%, Cat. #39220), and l-proline (≥99.0%, Cat. #P0380) were also purchased from Sigma-Aldrich (St. Louis, MO, USA). Whatman No. 4 filter paper was obtained from Whatman Intl., Ltd. (Maidstone, UK). Sodium carbonate (99.0%, Cat. #433401201), ether (99.0%, Cat. #33475S1280), and acetone (99.7%, Cat. #A0108) were obtained from Samchun Pure Chemical Co., Ltd. (Pyeongtaek, Korea).

The biogenic amine analysis extraction procedure was conducted as described by Kim and Ji [68]. Each 5 g sample was weighed and vortexed with 25 mL of 0.1 N HCl for 5 min. After the resulting homogenate was centrifuged at 10,000× *g* for 15 min at 4 °C (2236R high-speed centrifuge; Labogene Aps, Lillerød, Denmark), the aqueous layer was collected, and the residue was re-extracted as described above. The collected extracts were filtered through Whatman No. 4 filter paper. One milliliter of each extract was transferred to a glass test tube, and the following was added: 0.1 mL of internal standard (1,7-diaminoheptane, 100 mg/L), 0.5 mL of saturated sodium carbonate, and 1 mL of 1% dansyl chloride in acetone. After thoroughly mixing, the test tubes were incubated in a dark water bath (WBC 1510A; Jeio Tech. Co., Ltd., Seoul, Korea) at 45 °C for 60 min. Subsequently, 0.5 mL of 10% proline and 5 mL ether were added to each sample and allowed to rest for 5 min to remove the residual dansyl chloride. The supernatants were suspended and evaporated (Scanvac Speed Vacuum Concentrator; Labogene Aps, Lillerød, Denmark) at 20 °C until dry. The dry residue was diluted with 1 mL of acetonitrile (Sigma-Aldrich, St. Louis, MO, USA). The reconstituted sample and standard were filtered through a 0.2 µm syringe filter for HPLC analysis. The HPLC analysis of the biogenic amines was performed at the National Instrumentation Center for Environmental Management (NICEM) at Seoul National University (Seoul, Korea). The HPLC determinations were performed as described in Table 7.

### 3.5. Antimicrobial Susceptibility and Antibiotic Resistance Transferability Test

#### 3.5.1. Antimicrobial Agents

Twenty antimicrobial agents were used: ampicillin sodium salt (Sigma, Lot#BCBW1243), carbenicillin disodium salt (Sigma, Lot#116M4834V), cephalothin sodium salt (Sigma Lot#056M4858V), chloramphenicol (Sigma, Lot#SLBR8869V), clindamycin hydrochloride (Sigma, Lot#021M1533), dicloxacillin sodium salt hydrate (Sigma, Lot#SZBD263XV), erythromycin (Sigma, Lot#WXBC4044V), gentamicin sulfate (Sigma, Lot#SLBP3082V), kanamycin sulfate (Sigma, Lot#066M4019V), metronidazole (Sigma, Lot#MKBZ3056V), mupirocin (Sigma, Lot#106M4733V), neomycin sulfate (Sigma, Lot#LRAB3300), penicillin G (Sigma, Lot#087M4834V), phosphomycin disodium salt (Sigma, Lot#096M4031V), polymyxin B sulfate salt (Sigma, Lot#027M4002V), rifampicin (Sigma, Lot#MKCC2435), streptomycin sulfate salt (Sigma, Lot#SLBT8451), tetracycline (Sigma, Lot#126M4769V), trimethoprim–sulfamethoxazole (trimethoprim (Sigma, Lot#097M4017V), sulfamethoxazole (Sigma, Lot#BCBT3855)), vancomycin hydrochloride (USP, Lot#R07250). vancomycin hydrochloride was purchased from USP (Rockville, MD, USA), and the remaining 19 antimicrobiotics were purchased from Sigma (St. Louis, MO, USA). Each of the antibiotic powders was dissolved and diluted in appropriate diluents and filter sterilized prior to addition to LSM-Cys broth medium, composed of 90% of IST and 10% of MRS broth medium. IST broth was purchased from KisanBio Co., Ltd. (Mbcell Iso-Sensitest Broth, Seoul, Korea) and MRS was purchased from Becton, Dickinson and Company (BD Difco™ MRS Lactobacilli broth, Franklin Lakes, NJ, USA). Serial dilutions of antimicrobial agents ranging from 1024 to 0.0032 μg/mL were prepared.

#### 3.5.2. Antimicrobial Susceptibility Test

Minimal inhibitory concentration (MIC) values for all bacterial isolates were determined by the ISO 10932:2010 broth microdilution procedure [69]. The LSM-Cys broth medium supplemented with 0.03% (*w*/*v*) l-cysteine HCl containing antibiotics at different concentrations was used to prepare each well of a microwell plate. The inoculum was adjusted to a turbidity equivalent to 0.16 to 0.2 at 625 nm as measured by a Hitachi Spectrophotometer (Hitachi High-Technologies Co., Tokyo, Japan). The solution corresponded to approximately 3 × 10^8^ cfu/mL. Each inoculum was added to a double strength LSM-Cys broth medium at a rate of 0.2%. A 50 μL diluted bacterial suspension was added to each well; no negative control well was employed. The microdilution plates were prepared with a series of twofold dilutions of antibiotics. The microdilution plates were incubated at 37 °C for 48 h in an anaerobic (5% CO_2_, 10% H_2_ and 85% N_2_) chamber. The MIC was defined as the lowest concentration of antibiotic giving a complete inhibition of visible growth in comparison to an antibiotic-free control well. The experiments were replicated three times.

#### 3.5.3. Antibiotic Resistance Transferability Test

Conjugal transfer of antibiotic resistance was assessed via the methods of Tannock [70]. Equal bacterial cell volumes (1 mL) of the donor and recipient strains were mixed and centrifuged at 7000× *g* for 10 min (2236R high-speed centrifuge; Labogene Aps, Lillerød, Denmark) (see Table 8). After disposing of the supernatant, the bacterial cell pellet was resuspended in the MRS broth medium and cultivated at 37 °C for 12 h in an anaerobic chamber. The collected bacterial cells were filtered through a 0.45 μm microfilter membrane (Whatman Intl., Ltd., Maidstone, UK) and the membrane was placed on the surface of MRS agar and incubated anaerobically at 37 °C for 24 h. The bacterial cells were washed with 4 mL of 0.9% sterile saline, diluted to 10^−3^, 10^−4^, and 10^−5^, respectively, and then plated on MRS agar containing gentamicin or tetracycline. The plates were incubated aerobically or anaerobically at 37 °C for 36 h. Three replicates of all experiments were conducted.

#### 3.5.4. PCR Assay on Antibiotic Resistance Genes

The experimental conditions of Guo et al. [71] were used for these tests. The genomic DNA of the pure culture bacteria was extracted using MG™ Cell Genomic DNA Extraction SV miniprep (MGmed, Seoul, Korea). The extraction was performed according to the manufacturers’ instructions, and the total bacterial DNA was eluted with 200 μL of sterile water. To ensure that the ratio of absorbance at 260 nm to absorbance at 280 nm was 1.8–2.0., DNA extracts were aliquoted and stored at −20 °C. Polymerase chain reactions (PCR) were used to detect antibiotic resistance genes by gene-specific primers (Table 9). The following reaction mixture was added to each sample: 1.5 μL DNA (50 ng), 2 μL primer (100 pmol), dNTP mixture 8 μL, 2XGC buffer I, and adjusted to 50 μL volume by sterilized distilled water. The amplification program was an initial denaturation step of 94 °C for 5 min, and then 30 cycles of: 94 °C for 30 s, annealing temperature (Table 9) for 30 s, 72 °C for 1 min, and 72 °C for 7 min. The amplicons were analyzed on 1.5% agarose gel to confirm the DNA fragment size.

### 3.6. Mucin Degradation Test

Partially purified Mucin from porcine stomach—Type III, was purchased from Sigma (St. Louis, MO, USA). An MRS broth medium without a carbon source (i.e., basal medium containing yeast extract 0.75% (*w*/*v*), soy peptone 0.25% (*w*/*v*), fish extract 0.25% (*w*/*v*), sodium acetate 0.25% (*w*/*v*), ammonium citrate 0.1% (*w*/*v*), sodium phosphate monobasic 0.05% (*w*/*v*), sodium phosphate dibasic 0.025% (*w*/*v*), Tween 80 0.05% (*w*/*v*), l-cysteine HCl 0.05% (*w*/*v*), maleic acid 0.005% (*w*/*v*), taurine 0.00625% (*w*/*v*), magnesium sulfate 0.005% (*w*/*v*), manganese sulfate 0.0025% (*w*/*v*), and distilled water 98.2% (*v*/*v*)) was used as a negative control. To each of the four MRS broth media, 0.5% (*w*/*v*) mucin, 1.0% (*w*/*v*) mucin, 0.5% (*w*/*v*) glucose, and 1% (*w*/*v*) glucose were added. After the inoculation of the microorganisms in each MRS medium, the samples were cultured at 37 °C for 48 h under anaerobic conditions. After incubation, the bacterial growth was assessed by measuring absorbance at 550 nm at 12, 24, 36, and 48 h. The initial optical density value of the media was subtracted from the final value for each test sample.

### 3.7. Genetic Stability Test

*B. bifidum* BGN4 was plated on a MRS agar plate via streaking from a stock stored at −80 °C and incubated anaerobically at 37 °C for 24 h to obtain a single colony. A single colony was inoculated into 10 mL of MRS broth supplemented with 0.05% (*w*/*v*) l-cysteine HCl and regarded as the 2^0^ (1st) generation (about 10^6^ CFU/mL) of *B. bifidum* BGN4. *B. bifidum* BGN4 was incubated at 37 °C for about 12 h under anaerobic conditions to reach about 10^9^ cfu/mL and obtain 210 generations. In the second subculture, 0.1 mL (1% inoculation, about 10^6^ cfu/mL) of the primary culture was inoculated with 10 mL of MRS broth and cultured under the same conditions to obtain 220 generations of *B. bifidum* BGN4. For the third subculture, 0.1 mL (1% inoculation, approximately 10^6^ CFU/mL) of the secondary culture was inoculated with 10 mL of MRS broth and incubated to 10^7^ or 10^8^ CFU/mL to obtain 2^25^ generations of *B. bifidum* BGN4. The viable count during cultivation was measured to confirm the generation number. The genomic DNA of the pure culture bacteria was extracted using MG™ Cell Genomic DNA Extraction SV Miniprep (MGmed, Seoul, Korea), according to the manufacturer’s instructions. Whole genome sequencing and analysis were completed using an Illumina MiSeq sequencer and a Nextera XT Library Preparation kit (Illumina, San Diego, CA, USA). Nextera XT sequencing indices were used for multiplexing, and the participants were free to choose any sample index combination. The run acceptance criteria were a sequencing output of 5.6 Gb (to achieve an average sequencing coverage of 100-fold for the 20 samples with genome sizes of 2.8 Mb) and a Q30 read quality score of 75% [79]. The bioinformatics analysis was performed using Miseq raw data, and the comparative genomics analysis was completed with three Miseq raw data sets in ChunLab Co., Ltd. (Seoul, Korea).

### 3.8. Virulence Factors Researching

The search for virulence factors in *B. bifidum* BGN4 and *B. longum* BORI was completed using the VirulenceFinder1.5 Server, which is a component of the publicly available web-based tool for whole-genome sequencing(WGS) analysis hosted by the Center for Genomic Epidemiology (CGE) (http://www.genomicepidemiology.org/). The database system is designed to detect homologous sequences for the virulence genes related to *E. coli*, *Enterococcus*, *Listeria*, and *Staphylococcus aureus* in WGS data [80]. The output consists of best-matching genes from BLAST analysis of the selected database against the submitted genome of *B. bifidum* BGN4 or *B. longum* BORI. The selected %ID threshold was set at 90.00%, and the selected minimum length was set at 60%. If there is a matching result, the output shows information on the predicted virulence gene, the % ID, the length of query and database gene, the position of the hit in the contig, and the accession number of the hit.

## 4. Conclusions

Although probiotics have been widely used for their health benefits in food markets around the world, safety issues, including the side effects of probiotics, should be considered even more carefully than their clinical effects on consumers’ health. In this study, it is shown that *B. bifidum* BGN4 and *B. longum* BORI did not produce ammonia or biogenic amines such as histamine, tyramine, or cadaverine. A trace amount of putrescine was found in both strains; however, the quantities were similar to or less than the amount detected in various foods regularly consumed. Neither probiotic demonstrated hemolysis activity nor mucin degrading activity. Their resistance to antibiotics, however, was not transferable in this study. These finding suggest that *B. bifidum* BGN4 and *B. longum* BORI are suitable for use in foods with little risk of harmful effects on the consumer.

## Figures and Tables

**Table 1:** Mean value and standard deviation of ammonia level variables of *B. bifidum* BGN4, *B. longum* BORI, and other commercial microorganisms (*n* = 3).

Strain	Ammonia (μg/mL)
*Bifidobacterium bifidum* BGN4	negative
*Bifidobacterium longum* BORI	negative
*Bifidobacterium breve* ATCC 15701	negative
*Lactobacillus plantarum* KFRI 708	negative
*Bacteroides fragilis* ATCC 25285	14.7 ± 1.5
*Bacteroides thetaiotaomicron* ATCC 29741	23.3 ± 3.0
*Clostridium perfringens* ATCC 13124	23.5 ± 1.6
*Enterobacter cloacae* ATCC 13047	161.0 ± 6.6
*Enterobacter faecalis* ATCC 19433	12.9 ± 1.3

**Table 2:** Biogenic amine levels of *B. bifidum* BGN4 and *B. longum* BORI.

Strains	Cadaverine (µg/mL)	Histamine (µg/mL)	Putrescine (µg/mL)	Tyramine (µg/mL)
*B. bifidum* BGN4	N/D ^1^	N/D ^1^	24.23	N/D ^1^
*B. longum* BORI	N/D ^1^	N/D ^1^	16.58	N/D ^1^

^1^ N/D; not detected.

**Table 3:** Antimicrobial susceptibility (MIC values) of *B. bifidum* BGN4 and *B. longum* BORI and other *Bifidobacterium* spp.

Antibiotics	EFSA Cut-Off of *Bifidobacterium* spp.	*B. longum* ATCC 15707	*B. longum* BB536	*B. longum* KCCM 91563	*B. longum* BORI	*B. infantis* ATCC 15697	*B. lactis* BB-12	*B. bifidum* BGN4	*B. bifidum* KCTC 3440	*B. adolescentis* ATCC 15703	*B. breve* M-16V	*E. faecalis* ATCC 29212
Penicillin G		0.25	0.125	0.5	1	0.125	0.125	0.063	0.063	0.25	0.25	0.5
Carbenicillin disodium salt		2	2	4	8	0.5	2	0.5	0.5	4	4	8
Methicillin		8	4	16	16	1	2	1	0.5	8	8	16
Ampicillin sodium salt	2	0.5	0.25	1	0.5	0.125	0.125	0.063	0.063	0.25	0.25	0.25
Dicloxacillin sodium salt hydrate		4	4	8	8	0.5	4	0.5	1	256	8	4
Gentamicin sulfate	64	32	64	32	32	16	128	128	256	128	128	256
Streptomycin sulfate salt	128	32	128	64	64	>256	128	64	32	128	256	>256
Kanamycin sulfate	N/R ^1^	512	1024	1024	512	32	1024	1024	1024	1024	1024	256
Neomycin sulfate		1024	512	512	512	64	512	1024	512	512	1024	1024
Cephalothin sodium salt		8	4	16	32	4	8	4	2	16	16	16
Tetracycline	8	1	1	1	64	2	16	1	1	8	16	32
Polymyxin B sulfate salt		256	32	256	256	128	256	512	512	512	1024	>1024
Erythromycin	1	0.125	0.5	0.5	0.5	0.125	0.125	0.125	0.125	0.125	0.125	8
Metronidazole		8	8	>256	>256	8	4	4	64	>256	8	>256
Vancomycin hydrochloride	2	0.5	<0.25	<0.25	1	0.5	0.5	1	2	0.5	0.5	2
Chloramphenicol	4	2	2	2	4	2	2	2	2	2	2	8
Rifampicin		<0.125	<0.125	<0.125	0.25	<0.125	2	0.5	0.25	0.5	1	0.5
Clindamycin hydrochloride	1	<0.032	0.063	0.063	0.125	0.25	<0.032	0.063	0.063	<0.032	<0.032	>16
Phosphomycin disodium salt		128	256	256	256	16	64	128	256	64	32	32
Mupirocin		>128	>128	>128	>128	>128	>128	>128	>128	>128	>128	64
Trimethoprim–Sulfamethoxazole		128	256	128	256	256	1	128	64	1	2	32

^1^ N/R denotes not required.

**Table 4:** Transferability of tetracycline resistance from donors (*B. longum* BORI and *B. bifidum* BGN4) to recipients (*L. fermentum* AGBG1 and *L. acidophilus* ATCC 4356) (cfu/mL).

Antibiotics	AGBG1 (Aerobic)	AGBG1 + BORI		BORI (Anaerobic)
		Aerobic	Anaerobic	
None ^1^	4.38 × 10^8^	3.38 × 10^8^	2.27 × 10^8^	4.56 × 10^8^
T8 ^2^	0	0	4.44 × 10^6^	7.11 × 10^7^
Antibiotics	ATCC 4356 (Aerobic)	ATCC 4356 + BORI		BORI (Anaerobic)
		Aerobic	Anaerobic	
None ^1^	3.65 × 10^8^	1.67 × 10^8^	2.34 × 10^8^	3.14 × 10^8^
G64 ^3^	0	0	2.78 × 10^6^	1.46 × 10^8^
Antibiotics	ATCC 4356 (Aerobic)	ATCC 4356 + BGN4		BGN4 (Anaerobic)
		Aerobic	Anaerobic	
None ^1^	3.65 × 10^8^	3.29 × 10^8^	2.54 × 10^8^	3.86 × 10^8^
G64 ^3^	0	0	4.64 × 10^6^	1.43 × 10^8^

^1^ No antibiotics were included in the counting agar medium. ^2^ Tetracycline (8 μg/mL) was included in the counting agar medium. ^3^ Gentamicin (64 μg/mL) was included in the counting agar medium.

**Table 5:** OrthANI value

Strain/Sample	*B. bifidum* BGN4-1/13075.BBGN41.1 ^1^	*B. bifidum* BGN4-2/13075.BBGN42.1 ^2^	*B. bifidum* BGN4-3/13075.BBGN43.1 ^3^
*B. bifidum* BGN4-1/13075.BBGN41.1 ^1^	100	99.9997	99.9996
*B. bifidum* BGN4-2/13075.BBGN42.1 ^2^	99.9997	100	99.9998
*B. bifidum* BGN4-3/13075.BBGN43.1 ^3^	99.9996	99.9998	100

^1^*B. bifidum* BGN4-1/13075.BBGN41.1 denotes the 1st generation; ^2^ *B. bifidum* BGN4-2/13075.BBGN42.1 denotes the 25th generation; ^3^ *B. bifidum* BGN4-3/13075.BBGN43 and *B. bifidum* BGN4-2 are the 25th generations.

**Table 6:** Strain list and methods.

Strains	Origin	Medium	Method
*Bifidobacterium bifidum* BGN4	BIFIDO Co., Ltd. (Hongcheon, Korea)	BHI ^1^, Blood agar ^2^, whole milk ^3^, LSM-Cys ^4^, MRS ^5–8^	3.2., 3.3., 3.4., 3.5.2., 3.5.3., 3.5.4., 3.6., 3.7
*Bifidobacterium longum* BORI	BIFIDO Co., Ltd. (Hongcheon, Korea)	BHI ^1^, Blood agar ^2^, MRS ^3,5^^–^^7^, LSM-Cys ^4^	3.2., 3.3., 3.4., 3.5.2., 3.5.4., 3.6
*Bacteroides fragilis* ATCC 25285	American Type Culture Collection (Manassas, VA, USA)	BHI ^1^	3.2
*Bacteroides thetaiotaomicron* ATCC 29741	American Type Culture Collection (Manassas,VA, USA)	BHI ^1^	3.2
*Bifidobacterium adolescentis* ATCC 15703	American Type Culture Collection (Manassas,VA, USA)	LSM-Cys ^4^, MRS ^6^	3.5.2., 3.5.4
*Bifidobacterium animalis* ATCC 25527	American Type Culture Collection (Manassas,VA, USA)	MRS ^6^	3.5.4
*Bifidobacterium animalis* subsp. *lactis* AD011	BIFIDO Co., Ltd. (Hongcheon, Korea)	MRS ^6^	3.5.4
*Bifidobacterium animalis* subsp. *lactis* AS60	BIFIDO Co., Ltd. (Hongcheon, Korea)	MRS ^6^	3.5.4
*Bifidobacterium animalis* subsp. *lactis* BB-12	Isolated from a pharmaceutical product, USA	LSM-Cys ^4^, MRS ^6^	3.5.2., 3.5.4
*Bifidobacterium bifidum* KCTC 3440	Korean Collection for Type Cultures, (Jeongeup, Korea)	LSM-Cys ^4^, MRS ^6^	3.5.2., 3.5.4
*Bifidobacterium breve* ATCC 15701	American Type Culture Collection (Manassas,VA, USA)	BHI ^1^	3.2
*Bifidobacterium breve* M-16V	Isolated from a pharmaceutical product, USA	LSM-Cys ^4^, MRS ^6^	3.5.2., 3.5.4
*Bifidobacterium infantis* ATCC 15697	American Type Culture Collection (Manassas, VA, USA)	LSM-Cys ^4^, MRS ^6^	3.5.2., 3.5.4
*Bifidobacterium longum* ATCC 15707	American Type Culture Collection (Manassas,VA, USA)	LSM-Cys ^4^	3.5.2
*Bifidobacterium longum* BB536	Isolated from a pharmaceutical product, USA	LSM-Cys ^4^	3.5.2
*Bifidobacterium longum* KCCM 91563	Korean Culture Center of Microorganisms (Seoul, Korea)	LSM-Cys ^4^, MRS ^6^	3.5.2., 3.5.4
*Bifidobacterium longum* RD47	BIFIDO Co., Ltd. (Hongcheon, Korea)	MRS ^6^	3.5.4
*Bifidobacterium thermophilum* KCCM 12097	Korean Culture Center of Microorganisms (Seoul, Korea)	MRS ^6^	3.5.4
*Clostridium perfringens* ATCC 13124	American Type Culture Collection (Manassas,VA, USA)	BHI ^1^	3.2
*Enterococcus faecalis* ATCC 29212	American Type Culture Collection (Manassas,VA, USA)	LSM-Cys ^4^	3.5.2
*Enterobacter cloacae* subsp. *cloaca* ATCC 13047	American Type Culture Collection (Manassas,VA, USA)	BHI ^1^	3.2
*Enterobacter faecalis* ATCC 19433	American Type Culture Collection (Manassas,VA, USA)	BHI ^1^	3.2
*Lactobacillus acidophilus* ATCC 4356	American Type Culture Collection (Manassas,VA, USA)	MRS ^5^	3.5.3
*Lactobacillus fermentum* AGBG1	BIFIDO Co., Ltd. (Hongcheon, Korea)	MRS ^5^	3.5.3
*Lactobacillus plantarum* KFRI 708	Korea Food Research Institute (Wanju, Korea)	BHI ^1^	3.2
*Listeria ivanovii* subsp. *ivanovii* ATCC 19119	American Type Culture Collection (Manassas,VA, USA)	Blood Agar ^2^	3.3

^1^ Ammonia production test (3.2.): *B. bifidum* BGN4, *B. longum* BORI, *B. breve* ATCC 15701, *L. plantarum* KFRI 708, *B. fragilis* ATCC 25285, *B. thetaiotaomicron* ATCC 29741, *C. perfringens* ATCC 13124, *E. cloacae* ATCC 13047, and *E. faecalis* ATCC 19433 were anaerobically cultured in brain heart infusion (BHI) (BD BBL™, Franklin Lakes, NJ, USA) medium at 37 °C for 5 days. ^2^ Hemolytic test (3.3): *B. bifidum* BGN4 and *B. longum* BORI were anaerobically cultured in Blood agar (BHI broth medium supplemented with 1.5% agar and 5% sheep blood) at 37 °C for 2 days. *Listeria ivanovii* subsp. *ivanovii* ATCC 19119, a positive control for hemolysis, was aerobically cultivated in Blood agar at 37 °C for 2 days. ^3^ Biogenic amine production test (3.4): *B. bifidum* BGN4 and *B. longum* BORI, were anaerobically cultured in whole milk (Seoul Milk, Seoul, Korea) or de Man–Rogosa–Sharpe (MRS) broth (BD Difco™, Franklin Lakes, NJ, USA) with supplementation of 0.05% (*w*/*w*) l-cysteine-HCl (Sigma, St. Louis, MO, USA) at 37 °C for 15 h. ^4^ Antimicrobial susceptibility test (3.5.2.): LSM-Cys broth medium supplemented with 0.03% l-cysteine-HCl, which is composed with 90% of IST and 10% of MRS broth medium. ^5^ Antibiotic resistance transferability test (3.5.3.): *Bifidobacterium* strains were anaerobically cultured in MRS broth medium with supplementation of 0.05% (*w*/*v*) l-cysteine-HCl and *Lactobacillus* strains were cultured without l-cysteine-HCl at 37 °C for 18 h. ^6^ PCR assay on antibiotic resistance gene (3.5.4.): *Bifidobacterium* strains were anaerobically cultured in MRS broth medium with supplementation of 0.05% (*w*/*v*) l-cysteine-HCl at 37 °C for 18 h. ^7^ Mucin degradation test (3.6.): *B. bifidum* BGN4 and *B. longum* BORI were anaerobically cultured in MRS broth medium with supplementation of 0.05% (*w*/*v*) l-cysteine-HCl at 37 °C for 48 h. ^8^ Genetic stability test (3.7.): *B. bifidum* BGN4 was anaerobically cultured in MRS broth medium with supplementation of 0.05% (*w*/*v*) l-cysteine-HCl.

**Table 7:** HPLC conditions.

Parameters	Conditions		
HPLC	Thermo Dionex Ultimate 3000 HPLC (Thermo Fisher Scientific, St Peters, MO, USA)		
Column	VDSpher C-18 column (4.6 × 250 mm, 5 µm) (VDS optilab Chromatographietechnik GmbH, Berlin, Germany)		
Mobile solvent	Time (min)	Distilled Water (%)	Acetonitrile (%)
	0	40	60
	1	40	60
	20	0	100
	25	0	100
	26	40	60
	30	40	60
Flow rate	0.8 mL		
Column temperature	30 °C		
Injection volume	20 µL		
Detector	UV 250 nm		

**Table 8:** Test scheme.

Donor Strains Recipient Strains	*B. bifidum* BGN4	*B. longum* BORI
*L. fermentum* AGBG1	N/A ^1^	BORI + AGBG1
*L. acidophilus* ATCC 4356	BGN4 + ATCC 4356	BORI + ATCC 4356

^1^ N/A denotes not applicable because *B. bifidum* BGN4 was highly susceptible to tetracycline, which resulted in no growth on the media containing tetracycline.

**Table 9:** Primers and conditions for PCR detection ^1^.

No.	Primer Name		Oligo Sequence	TM (°C)	Product Size	Reference
1	*Bifidobacterium* genus-specific primers	-	F: 5′-TCGCGTCYGGTGTGAAAG-3′ R: 5′-GGTGTTCTTCCCGATATCTACA-3′	55	128 bp	[72]
2	Gentamicin	*aaac(6)-aph(2)*	F: 5′-CCAAGAGCAATAAGGGCATA-3′ R: 5′-CACTATCATAACCACTACCG-3′	60	220 bp	[73]
3	Kanamycin	*AphA3*	F: 5′-GCCGATGTGGATTGCGAAAA-3′ R: 5′-GCTTGATCCCCAGTAAGTCA-3′	52	292 bp	[74]
4	Streptomycin	*aadE*	F: 5′-ATGGAATTATTCCCACCTGA-3′ R: 5′-TCAAAACCCCTATTAAAGCC-3′	50	565 bp	[74]
5	Trimethoprim	*dfrA*	F: 5′-AAAAGGGGCAGAGCATG-3′ R: 5′-AGAAAATGGCGTAATCGGTA-3′	50	474 bp	[75]
6	Tetracycline(K)	*tet*(K)	F: 5′-TTAGGTGAAGGGTTAGGTCC-3′ R: 5′-GCAAACTCATTCCAGAAGCA-3′	55	169 bp	[76]
7	Tetracycline(L)	*tet*(L)	F: 5′-GTTGCGCGCTATATTCCAAA-3′ R: 5′-TTAAGCAAACTCATTCCAGC-3′	55		
8	Tetracycline(M)	*tet*(M)	F: 5′-GTTAAATAGTGTTCTTGGAG-3′ R: 5′-CTAAGATATGGCTCTAACAA-3′	55	401 bp	[77]
9	Tetracycline(O)	*tet*(O)	F: 5′-GATGGCATACAGGCACAGAC-3′ R: 5′-CAATATCACCAGAGCAGGCT-3′	55		
10	Tetracycline(S)	*tet*(S)	F: 5′-TGGAACGCCAGAGAGGTATT-3′ R: 5′-ACATAGACAAGCCGTTGACC-3′	55	1923 bp	[78]
11	Kanamycin	*aaaD*	F: 5′-TGCGTTTTGACACATCCAC-3′ R: 5′-GGTGTTTATGGCTCTCTTGG-3′	55		

^1^ The experiment conditions are secondary quoted from Guo et al. [71].

## References

[1] Lilly D.M., Stillwell R.H. (1965). Probiotics: Growth-Promoting Factors Produced by Microorganisms. Science.

[2] Probiotics: In Depth. https://nccih.nih.gov/health/probiotics/introduction.htm.

[3] Food and Agriculture Organization-World Health Organization (FAO/WHO) (2002). Report on Joint FAO/WHO Guidelines for the Evaluation of Probiotics in Food. http://www.who.int/foodsafety/fs_management/en/probiotic_guidelines.pdf.

[4] European Food and Feed Cultures Association (EFFCA) (2003). Definition of Microbial Food Culture (MFC).

[5] Akhter N., Wu B., Memon A.M., Mohsin M. (2015). Probiotics and Prebiotics Associated with Aquaculture: A Review. Fish Shellfish Immunol..

[6] Ford A.C., Quigley E.M., Lacy B.E., Lembo A.J., Saito Y.A., Schiller L.R., Soffer E.E., Spiegel B.M., Moayyedi P. (2014). Efficacy of Prebiotics, Probiotics and Synbiotics in Irritable Bowel Syndrome and Chronic Idiopathic Constipation: Systematic Review and Meta-Analysis. Am. J. Gastroenterol..

[7] O’Hara A.M., Shanahan F. (2006). The Gut Flora as a Forgotten Organ. EMBO Rep..

[8] Sánchez B. (2018). Bile Acid–Microbiota Crosstalk in Gastrointestinal Inflammation and Carcinogenesis: A Role for Bifidobacteria and Lactobacilli?. Nat. Rev. Gastroenterol Hepatol..

[9] Lukjancenko O., Ussery D.W., Wassenaar T.M. (2012). Comparative genomics of *Bifidobacterium*, *Lactobacillus* and Related Probiotic Genera. Microb. Ecol..

[10] Global Market Insights Inc Probiotics Market Size to Exceed USD 64 Billion by 2023: Global Market Insights Inc. https://www.prnewswire.com/news-releases/probiotics-market-size-to-exceed-usd-64-billion-by-2023-global-market-insights-inc-578769201.html.

[11] EFSA Update of the List of QPS-Recommended Biological Agents Intentionally Added to Food or Feed as Notified to EFSA 4: Suitability of Taxonomic Units Notified to EFSA until March 2016. https://www.efsa.europa.eu/en/efsajournal/pub/4522.

[12] Anadón A., Martínez-Larrañaga M.R., Ares I., Martínez M.A., Hill-Parks E. (2016). Nutraceuticals; Efficacy, Safety and Toxicity. Chapter 55—Probiotics: Safety and Toxicity Considerations.

[13] Huys G., Botteldoorn N., Delvigne F., de Vuyst L., Heyndrickx M., Pot B., Dubois J.J., Daube G. (2013). Microbial Characterization of Probiotics-Advisory Report of the Working Group “8651 Probiotics” of the Belgian Superior Health Council (SHC). Mol. Nutr. Food Res..

[14] Shokryazdan P., Jahromi M.F., Liang J.B., Kalavathy R., Sieo C.C., Ho Y.W. (2016). Safety Assessment of Two New Lactobacillus Strains as Probiotic for Human Using a Rat Model. PLoS ONE.

[15] Tan Q., Xu H., Aguilar Z.P., Peng S., Dong S., Wang B., Li P., Chen T., Xu F., Wei H. (2013). Safety Assessment and Probiotic Evaluation of *Enterococcus faecium* Isolated from Sourdough. J. Food Sci..

[16] Endres J.R., Clewell A., Jade K.A., Farber T., Hauswirth J., Schauss A.G. (2009). Safety Assessment of a Proprietary Preparation of a Novel Probiotic, *Bacillus coagulans*, as a Food Ingredient. Food Chem. Toxicol..

[17] Sanders M.E., Akkermans L.M.A., Haller D., Hammerman C., Heimbach J., Hörmannsperger G., Huys G., Levy D.D., Lutgendorff F., Mack D. (2010). Safety Assessment of Probiotics for Human Use. Gut Microbes..

[18] Salyers A.A., Gupta A., Wang Y. (2004). Human Intestinal Bacteria as Reservoirs for Antibiotic Resistance Genes. Trends Microbiol..

[19] Sommer M.O.A., Dantas G., Church G.M. (2009). Functional Characterization of the Antibiotic Resistance Reservoir in the Human. Science.

[20] Fouhy F., Guinane C.M., Hussey S., Wall R., Ryan C.A., Dempsey E.M., Murphy B., Ross R.P., Fitzgerald G.F., Stanton C. (2012). High-Throughput Sequencing Reveals the Incomplete, Short-Term, Recovery of the Infant Gut Microbiota Following Parenteral Antibiotic Treatment with Ampicillin and Gentamicin. Antimicrob. Agents. Chemother..

[21] Fallani M., Young D., Scott J., Norin E., Amarri S., Adam R., Aguilera M., Khanna S., Gil A., Edwards C.A. (2010). Intestinal microbiota of 6-week-old infants across Europe: Geographic influence beyond delivery mode, breast-feeding, and antibiotics. J. Pediatr. Gastroenterol. Nutr..

[22] Murphy E.F., Cotter P.D., Healy S., Marques T.M., O’Sullivan O., Fouhy F., Clarke S.F., O’Toole P.W., Quigley E.M., Stanton C. (2010). Composition and energy harvesting capacity of the gut microbiota: Relationship to diet, obesity and time in mouse models. Gut.

[23] Imperial I.C.V.J., Ibana J.A. (2016). Addressing the Antibiotic Resistance Problem with Probiotics: Reducing the Risk of Its Double-Edged Sword Effect. Front. Microbiol..

[24] O’Callaghan A., van Sinderen D. (2016). Bifidobacteria and Their Role as Members of the Human Gut Microbiota. Front. Microbiol..

[25] Park M.S., Kwon B., Ku S., Ji G.E. (2017). The Efficacy of *Bifidobacterium longum* BORI and *Lactobacillus acidophilus* AD031 Probiotic Treatment in Infants with Rotavirus Infection. Nutrients.

[26] Seo J.M., Ji G.E., Cho S.H., Park M.S., Lee H.J. (2007). Characterization of a *Bifidobacterium longum* BORI Dipeptidase Belonging to the U34 Family. Appl. Environ. Microbiol..

[27] JI G.E. (2005). Development of *Bifidobacterium* sp. BGN4 and BORI with Novel Probiotic Activity. Int. Meet. Microbiol. Soc. Korea.

[28] Ku S., Park M.S., Ji G.E., You H.J. (2016). Review on *Bifidobacterium bifidum* BGN4: Functionality and Nutraceutical Applications as a Probiotic Microorganism. Int. J. Mol. Sci..

[29] Ku S., You H.J., Ji G.E. (2009). Enhancement of Anti-Tumorigenic Polysaccharide Production, Adhesion, and Branch Formation of *Bifidobacterium bifidum* BGN4 by Phytic Acid. Food Sci. Biotechnol..

[30] Health Functional Food Code (HFFC) (2010). II.2.51. Probiotics. Ministry of Food and Drug Safety in Korea. http://www.mfds.go.kr/files/upload/eng/4.Health_Functioanl_Food_Code_(2010.09).pdf.

[31] Yu D.S., Jeong H., Lee D.H., Kwon S.K., Song J.Y., Kim B.K., Park M.S., Ji G.E., Oh T.K., Kim J.F. (2012). Complete Genome Sequence of the Probiotic Bacterium *Bifidobacterium bifidum* strain BGN4. J. Bacteriol..

[32] Igai K., Itakura M., Nishijima S., Tsurumaru H., Suda W., Tsutaya T., Tomitsuka E., Tadokoro K., Baba J., Odani S. (2016). Nitrogen Fixation and *nif*H Diversity in Human Gut Microbiota. Sci. Rep..

[33] Smith E.A., Macfarlane G.T. (1997). Formation of Phenolic and Indolic Compounds by Anaerobic Bacteria in the Human Large Intestine. Microbiol. Ecol..

[34] Vince A.J., Burridge S.M. (1980). Ammonia Production by Intestinal Bacteria: The Effects of Lactose, Lactulose and Glucose. J. Med. Microbiol..

[35] Zarei M., Najafzadeh H., Enayati A., Pashmforoush M. (2011). Biogenic Amines Content of Canned Tuna Fish Marketed in Iran. Am.-Eurasian J. Toxicol. Sci..

[36] Ku S. (2016). Finding and Producing Probiotic Glycosylases for the Biocatalysis of Ginsenosides: A Mini Review. Molecules.

[37] Biji K.B., Ravishankar C.N., Venkateswarlu R., Mohan C.O., Srinivasa Gopal T.K. (2016). Biogenic Amines in Seafood: A review. J. Food Sci. Technol..

[38] Jansen S.C., van Dusseldorp M., Bottema K.C., Dubois A.E. (2003). Intolerance to Dietary Biogenic Amines: A Review. Ann. Allergy Asthma Immunol..

[39] Burdychová R., Komprda T. (2007). Biogenic Amine-Forming Microbial Communities in Cheese. FEMS Microbiol. Lett..

[40] Priyadarshani W.M.D., Rakshit S.K. (2011). Screening Selected Strains of Probiotic Lactic Acid Bbacteria for Their Ability to Produce Biogenic Amines (Histamine and Tyramine). Int. J. Food. Sci. Technol..

[41] Lorencová E., Buňková L., Matoulková D., Dráb V., Pleva P., Kubáň V., Buňká F. (2012). Production of Biogenic Amines by Lactic Acid Bacteria and Bifidobacteria Isolated from Dairy Products and Beer. Int. J. Food. Sci. Technol..

[42] Kalač P., Švecová S., Pelikánová T. (2002). Levels of Biogenic Amines in Typical Vegetable Products. Food Chem..

[43] Ten Brink B., Damink C., Joosten H.M.L.J., Huis in’t Veld J.H.J. (1990). Occurrence and Formation of Biologically Active Amines in Foods. Int. J. Food Microbiol..

[44] Manca G., Porcu A., Ru A., Salaris M., Franco M.A., De Santis E.P.L. (2015). Comparison of γ-Aminobutyric Acid and Biogenic Amine Content of Different Types of Ewe’s Milk Cheese Produced in Sardinia. Ital. J. Food Saf..

[45] Pollack P.F., Koldovskỳ O., Nishioka K. (1992). Polyamines in Human and Rat Milk and in the Infant Formulas. Am. J. Clin. Nutr..

[46] EFSA Panel on Biological Hazards (BIOHAZ) (2011). Scientific Opinion on Risk Based Control of Biogenic Amine Formation in Fermented Foods. EFSA J..

[47] Borriello S.P., Hammes W.P., Holzapfel W., Marteau P., Schrezenmeir J., Vaara M., Valtonen V. (2003). Safety of Probiotics That Contain Lactobacilli or Bifidobacteria. Clin. Infect. Dis..

[48] Ammor M.S., Flórez A.B., Mayo B. (2007). Antibiotic Resistance in Non-Enterococcal Lactic Acid Bacteria and Bifidobacteria. Food Microbiol..

[49] EFSA Panel on Additives and Products or Substances used in Animal Feed (FEEDAP) (2012). Guidance on the Assessment of Bacterial Susceptibility to Antimicrobials of Human and Veterinary Importance. EFSA J..

[50] Flórez A.B., Ammor M.S., Mayo B., van Hoek A.H.A.M., Aarts H.J.M., Huys G. (2008). Antimicrobial Susceptibility Profiles of 32 Type Strains of *Lactobacillus*, *Bifidobacterium*, *Lactococcus* and *Streptococcus* spp.. Int. J. Antimicrob. Agents.

[51] Georgieva R., Yochevab L., Tserovskab L., Zhelezovab G., Stefanovaa N., Atanasovaa A., Dangulevaa A., Ivanovaa G., Karapetkova N., Rumya N. (2015). Antimicrobial Activity and Antibiotic Susceptibility of *Lactobacillus* and *Bifidobacterium* spp. Intended for Use as Starter and Probiotic Cultures. Biotechnol. Biotechnol. Equip..

[52] Duranti S., Lugli G.A., Mancabelli L., Turroni F., Milani C., Mangifesta M., Ferrario C., Anzalone R., Viappiani A., van Sinderen D. (2017). Prevalence of Antibiotic Resistance Genes among Human Gut-Derived Bifidobacteria. Appl. Environ. Microbiol..

[53] GRAS notice (GRN) No. 685 Generally Recognized as Safe (GRAS) Determination for the Use of Lactobacillus plantarum 299v in Conventional Foods. https://www.fda.gov/downloads/Food/IngredientsPackagingLabeling/GRAS/NoticeInventory/ucm544492.pdf.

[54] Mättö J., van Hoek A.H.A.M., Domig K.J., Saarela M., Floréz A.B., Brockmann E., Amtmann E., Mayo B., Aarts H.J.M., Danielsen M. (2007). Susceptibility of Human and Probiotic *Bifidobacterium* spp. to Selected Antibiotics as Determined by the Etest Method. Int. Dairy J..

[55] Lim K.S., Huh C.S., Baek Y.J.A. (1995). Selective Enumeration Medium for Bifidobacteria in Fermented Dairy Products. J. Dairy Sci..

[56] (2018). Food Code (2018-8). 7. General Test Methods: 4.4.1 Medium 25) TOS-MUP Medium; 4.9.2 Acid-Bacterial Streptococcus and Bifidus. Ministry of Food and Drug Safety in Korea. http://www.foodsafetykorea.go.kr/portal/safefoodlife/food/foodRvlv/foodRvlv.do.

[57] National Food Safety Standard (2016). Microbiological Examination of Food—Examination of Lactic Acid Bacteria(GB4789.35-2016) 4. Medium and Reagents 4.2 Medium and Reagents.

[58] Agency for Healthcare Research and Quality Advancing Excellence in Health Care (AHRQ) (2011). Safety of Probiotics to Reduce Risk and Prevent or Treat Disease.

[59] Fouhy F., Motherway M.O., Fitzgerald G.F., Ross R.P., Stanton C., van Sinderen D., Cotter P.D. (2013). In Silico Assig ned Resistance Genes Confer *Bifidobacterium* with Partial Resistance to Aminoglycosides but Not to β-Lactams. PLoS ONE.

[60] Gueimonde M., Sánchez B., Reyes-Gavilán C.G., Margolles A. (2013). Antibiotic Resistance in Probiotic Bacteria. Front. Microbiol..

[61] UniProt UniProtKB-O52836 (TETW_BUTFI). http://www.uniprot.org/uniprot/O52836.

[62] De Groote M.A., Frank D.N., Dowell E., Glode M.P., Pace N.R. (2005). *Lactobacillus rhamnosus* GG Bacteremia Associated with Probiotic Use in a Child with Short Gut Syndrome. Pediatr. Infect. Dis. J..

[63] Liong M.T. (2008). Safety of Probiotics: Translocation and Infection. Nutr. Rev..

[64] Ruas-Madiedo P., Gueimonde M., Fernández-García M., de los Reyes-Gavilán C.G., Margolles A. (2008). Mucin Degradation by *Bifidobacterium* Strains Isolated from the Human Intestinal Microbiota. Appl. Environ. Microbiol..

[65] Ruseler-van Embden J.G., Liesholt L.M., Gosselink M.J., Marteau P. (1995). Inability of *Lactobacillus casei* Strain GG, *L. acidophilus* and *Bifidobacterium bifidum* to Degrade Intestinal Mucus Glycoproteins. Scand. J. Gastroenterol..

[66] Abe F., Muto M., Yaeshima T., Iwatsuki K., Aihara H., Ohashi Y., Fujisawa T. (2010). Safety Evaluation of Probiotic Bifidobacteria by Analysis of Mucin Degradation Activity and Translocation Ability. Anaerobe.

[67] Chaney A.L., Marbach E.P. (1962). Modified Reagents for Determination of Urea and Ammonia. Clin. Chem..

[68] Kim N.Y., Ji G.E. (2015). Characterization of the Production of Biogenic Amines and Gamma-Aminobutyric Acid in the Soybean Pastes Fermented by *Aspergillus oryzae* and *Lactobacillus brevis*. J. Microbiol. Biotechnol..

[69] International Organization for Standardization (ISO) (2010). Milk and Milk Products-Determination of the Minimal Inhibitory Concentration (MIC) of Antibiotics Applicable to Bifidobacteria and Non-Enterococcal Lactic Acid Bacteria (LAB).

[70] Tannock G.W. (1987). Conjugal Transfer of Plasmid pAMßi in *Lactobacillus reuteri* and between Lactobacilli and *Enterococcus faecalis*. Appl. Environ. Microbiol..

[71] Guo H.L., Pan L., Li L.N., Lu J., Kwok L., Menghe B., Zhang H.P., Zhang W.Y. (2017). Characterization of Antibiotic Resistance Genes from *Lactobacillus* Isolated from Traditional Dairy Products. J. Food Sci..

[72] Thapa D., Louis P., Losa R., Zweifel B., Wallace R.J. (2015). Essential oils have different effects on human pathogenic and commensal bacteria in mixed faecal fermentations compared with pure cultures. Microbiology..

[73] Rojo-Bezares B., Saenz Y., Poeta P., Zarazaga M., Ruiz-Larrea F., Torres C. (2006). Assessment of antibiotic susceptibility within lactic acid bacteria strains isolated from wine. Int. J. Food Microbiol..

[74] Ouoba L.I., Lei V., Jensen L.B. (2008). Resistance of potential probiotic lactic acid bacteria and bifi-dobacteria of African and European origin to antimicrobials: Determination and transferability of the resistance genes to other bacteria. Int. J. Food Microbiol..

[75] Liu C., Zhang Z.Y., Dong K., Yuan J.P., Guo X.K. (2009). Antibiotic resistance of probiotic strains of lactic acid bacteria isolated from marketed foods and drugs. Biomed Environ Sci..

[76] Aquilanti L., Garofalo C., Osimani A., Silvestri G., Vignaroli C., Clementi F. (2007). Isolation and molecular characterization of antibiotic-resistant lactic acid bacteria from poultry and swine meat products. J. Food. Prot..

[77] Gad G.F., Abdel-Hamid A.M., Farag Z.S. (2014). Antibiotic resistance in lactic acid bacteria isolated from some pharmaceutical and dairy products. Braz. J. Microbiol..

[78] Charpentier E., Gerbaud G., Courvalin P. (1993). Characterization of a new class of tetracycline-resistance gene tet(S) in *Listeria monocytogenes* BM4210. Gene.

[79] Mellmann A., Andersen P.S., Bletz S., Friedrich A.W., Kohl T.A., Lilje B., Niemann S., Prior K., Rossen J.W., Harmsen D. (2017). High Interlaboratory Reproducibility and Accuracy of Next-Generation-Sequencing-Based Bacterial Genotyping in a Ring Trial. J. Clin. Microbiol..

[80] Joensen K.G., Scheutz F., Lund O., Hasman H., Kaas R.S., Nielsen E.M., Aarestrup F.M. (2014). Real-time whole-genome sequencing for routine typing, surveillance, and outbreak detection of verotoxigenic *Escherichia coli*. J. Clin. Micobiol..

